# Antibacterial Activities and Possible Modes of Action of *Acacia nilotica* (L.) Del. against Multidrug-Resistant *Escherichia coli* and *Salmonella*

**DOI:** 10.3390/molecules22010047

**Published:** 2017-01-14

**Authors:** Muhammad Bilal Sadiq, Joel Tarning, Tay Zar Aye Cho, Anil Kumar Anal

**Affiliations:** 1 Food Engineering and Bioprocess Technology, Asian Institute of Technology, Bangkok 12120, Thailand; m.bilalsadiq@hotmail.com (M.B.S.); actayxar@gmail.com (T.Z.A.C.); 2 Mahidol-Oxford Tropical Medicine Research Unit, Faculty of Tropical Medicine, Mahidol University, Bangkok 10400, Thailand; joel@tropmedres.ac; 3 Centre for Tropical Medicine and Global Health, Nuffield Department of Clinical Medicine, University of Oxford, Oxford OX3 7FZ, UK

**Keywords:** antibacterial activity, antibiogram, kill-time analysis, SEM, bacterial membrane permeability

## Abstract

Medicinal plants are frequently used for the treatment of various infectious diseases. The objective of this study was to evaluate the antibacterial activity and mode of action of *Acacia nilotica* and the antibiogram patterns of foodborne and clinical strains of *Escherichia coli* and *Salmonella*. The mechanism of action of acacia extracts against *E. coli* and *Salmonella* was elucidated by observing morphological damages including cell integrity and cell membrane permeability, as well as changes in cell structures and growth patterns in kill-time experiments. The clinical isolates of *E. coli* and *Salmonella* were found resistant to more of the tested antibiotics, compared to food isolates. Minimum inhibitory concentration and minimum bactericidal concentration of acacia leaf extracts were in the ranges of 1.56–3.12 mg/mL and 3.12–6.25 mg/mL, respectively, whereas pods and bark extracts showed somewhat higher values of 3.12–6.25 mg/mL and 6.25–12.5 mg/mL, respectively, against all tested pathogens. The release of electrolytes and essential cellular constituents (proteins and nucleic acids) indicated that acacia extracts damaged the cellular membrane of the pathogens. These changes corresponded to simultaneous reduction in the growth of viable bacteria. This study indicates that *A. nilotica* can be a potential source of new antimicrobials, effective against antibiotic-resistant strains of pathogens.

## 1. Introduction

Though antibiotics provide the main basis for treatment of bacterial infections, emerging bacterial resistance to commonly used antibiotics is a global concern. Repeated exposure and overuse of antibiotics have led to an increasing rate of antibacterial resistance and the development of multidrug-resistant strains of microorganisms [1]. Antimicrobial resistance monitoring is essential for providing information on the magnitude and trends of microbial resistance in order to plan and monitor the effect of targeted interventions [2].

*Salmonella* and *Escherichia coli* are included in the main food-borne pathogens responsible for food poisoning and subsequent enteric infections [3,4]. In South East Asia, there is an absence of official *Salmonella* surveillance, but it is estimated that up to 22.8 million cases of salmonellosis occur annually with 37,600 deaths [5]. *Salmonella* and *E. coli* isolated from poultry meat were found resistant to various commercially available antibiotics [6,7]. Food contaminated with drug-resistant bacteria is a major threat to public health [8].

Thus, there is an urgent need of new antimicrobial agents to combat the rapid emergence of bacterial resistance. However, the rapid widespread resistance development indicates that even new antimicrobial agents will have a relatively short therapeutic life span [9]. An underappreciated source of novel antimicrobial agents with potentially new mechanism of actions might be natural plant sources and herbal products [10]. Plants contain secondary metabolites in addition to minerals and primary metabolites that are responsible for antioxidant and antibacterial effects [11].

The use of medicinal plants as an alternative therapy against various infectious diseases is age-long practice. This might be best exemplified in the field of antimalarial therapy, where the recommended first-line active drug compound (i.e., artemisinins) was originally derived from *Artemisia annua*. *Acacia nilotica* (L.) Del. is a medicinal plant belonging to the family *Mimosaceae*. The plant is widely distributed in tropical and subtropical regions. Ayurvedic medicine practices suggest the use of leaves, bark and pods of *A. nilotica* against cancer, cough, diarrhea, fever, small pox, piles and menstrual problems [12]. The plant is reported to have antibacterial effects against pathogenic microorganisms such as *Mycobacterium tuberculosis*, *Pseudomonas aeruginosa*, *Escherichia coli* and *Staphylococcus aureus* [13]. *A. nilotica* showed high antimicrobial potential against *Staphylococcus aureus*, *E. coli* and *Salmonella typhi* in a comparative antimicrobial study among acacia species [14]. Ethanol and petroleum ether extracts of *A. nilotica* displayed antibacterial effects against *S. aureus, E. coli*, *Proteus vulgaris*, *Proteus mirabilis*, *Salmonella paratyphi* and *Klebsiella pneumoniae* [15]. A number of researchers have investigated the antimicrobial activity of *A. nilotica*. However, the mechanism of action has not been studied in detail. Thus, the objective of this study was to evaluate the antimicrobial effects and possible antimicrobial modes of action of *A. nilotica* leaves, pods and bark extracts against multidrug-resistant strains of *E. coli* and *Salmonella*.

## 2. Results and Discussion

### 2.1. Antibiogram of Salmonella and E. coli

The prevalence of antibacterial drug resistance is high among foodborne pathogens due to extensive therapeutic and prophylactic use of antibiotics in animal farming. In South East Asia, multidrug-resistant strains of *E. coli* and *Salmonella* are frequently isolated from food sources. The treatment of infections caused by multidrug-resistant bacteria is becoming an increasingly more difficult task and is of great food and patient safety concern [16,17].

Antibiogram patterns of bacterial isolates were determined by the disc diffusion assay, following the guidelines of Clinical and Laboratory Standard Institute (CLSI). The results were interpreted by measuring the diameter of inhibition zone. The antibacterial susceptibility testing of *E. coli* and *Salmonella* spp. obtained from clinical and food sources demonstrated that both clinical and food source isolates of *E. coli* and *Salmonella* were resistant to various antibiotics as shown in Table 1. *E. coli* (E1) obtained from human clinical isolates was found to be resistant to ampicillin (10 µg), amoxicillin (10 µg), chloramphenicol (30 µg), tetracycline (30 µg), ciprofloxacin (5 µg) and ceftriaxone (30 µg), whereas clinical isolate of *Salmonella typhimurium* (S1) was found to be resistant to ampicillin (10 µg), amoxicillin (10 µg), tetracycline (30 µg) and streptomycin (10 µg). *E. coli* and *Salmonella enterica* (E2 and S2, respectively) isolated from poultry meat were found to be susceptible to all the tested antibiotics, whereas *E. coli* (E3), isolated from beef meat was resistant to ampicillin (10 µg), amoxicillin (10 µg), chloramphenicol (30 µg) and tetracycline (30 µg); *Salmonella typhimurium* (S3), isolated from poultry meat was resistant to tetracycline (30 µg), and chloramphenicol (30 µg); *Salmonella enteritidis* (S4), isolated from beef meat was resistant to ampicillin (10 µg), amoxicillin (10 µg) and tetracycline (30 µg).

The antibiogram of tested pathogens demonstrated that the clinical isolates of *E. coli* (E1) and *Salmonella* (S1) were highly resistant, which might be due to previous exposure of pathogens to antibiotics and their ability to develop resistance upon repeated exposure in humans. Moreover, both strains were found to be resistant to beta-lactam antibiotics including ampicillin (10 µg) and amoxicillin (10 µg) indicating the ability to produce beta-lactamase. These results support a previous report, suggesting that *E. coli* isolated from various meat samples were found resistant to beta-lactams and tetracycline antibiotics [18].

### 2.2. Detection of Beta-Lactams- and Tetracycline-Resistant Genes

**Figure 1:**
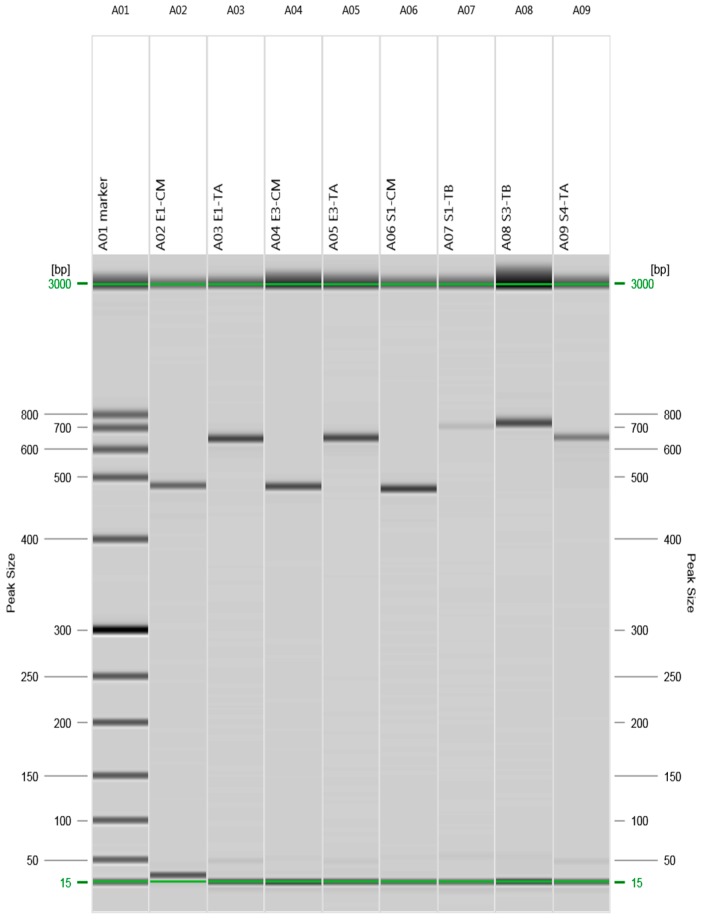
E1 = *E. coli* and S1 = *Salmonella typhimurium* were isolated from clinical samples, S3 = *Salmonella typhimurium* was isolated from poultry meat, and E3 = *E. coli* and S4 = *Salmonella enteritidis* were isolated from beef meat samples. CM represents beta-lactam-resistant gene and TA and TB represent tetracycline-resistant genes.

The presence of antibiotic resistance genes encoding resistance to the beta-lactam and tetracycline class of antibiotics was investigated using polymerase chain reaction (PCR) with genomic DNA, and amplified products were resolved by gel electrophoresis (Figure 1).

*E. coli* (E1) obtained from human clinical isolates showed the presence of *bla _CMY_* and *tet (A)* genes, whereas clinical isolate of *Salmonella typhimurium* (S1) showed the presence of *bla _CMY_* and *tet (B)*. *E. coli* (E3) isolated from beef meat showed the presence of *bla _CMY_* and *tet (A)*; *Salmonella typhimurium* (S3) isolated from poultry meat and *Salmonella enteritidis* (S4) isolated from beef meat showed the presence of *tet (B)* and *tet (A)*, respectively. *E. coli* and *Salmonella enterica* (E2 and S2, respectively) isolated from poultry meat were found negative for all the tested resistant genes and this finding was in accordance with the antibiotic susceptibility results.

### 2.3. Antimicrobial Activity of Acacia Extracts

The antimicrobial activities of acacia extracts were investigated by the disc diffusion assay at different concentrations (5 and 10 mg/disc). The results were read after 24 h of incubation at 37 °C by measuring the diameter of inhibition zone (Table 2). All selected parts of acacia were found to be effective against the selected pathogens. The lowest tested concentration (5 mg/disc) of all extracts of acacia inhibited the growth of both clinical and food isolates of *E. coli* and *Salmonella*. Similar results were previously reported by Kavitha et al. [19], who studied the antibacterial effects of *A. nilotica* against various clinical bacterial isolates. The leaf extracts at a concentration of 10 mg/disc showed the maximum mean diameter zone of inhibition of 21.11 ± 1.05 mm and 16.83 ± 0.94 mm against *E. coli* and *Salmonella* strains, respectively. The leaves were found more effective in inhibiting bacterial growth as compared to pods and bark extracts. The results demonstrated that all selected pathogens were susceptible to all tested parts of the plant. This indicated a strong antibacterial potential of *A. nilotica* against the antibiotic-resistant pathogens tested here, arguably with a novel mechanism of action than other tested antibiotics. The antibacterial results of the current study are in accordance with literature, where extracts of *A. nilotica* were effective against clinical bacterial isolates of *E. coli* and *Salmonella* [20,21].

### 2.4. Minimum Inhibitory Concentration and Minimum Bactericide Concentration of Acacia nilotica Extracts

Minimum inhibitory concentration (MIC) values of acacia extracts were determined by broth macro-dilution method and minimum bactericide concentration (MBC) values were estimated by sub-culturing all concentrations (≥MIC) that had no detectable growth. MIC and MBC of acacia leaves were in the range of 1.56–3.12 mg/mL and 3.12–6.25 mg/mL, respectively, against all tested bacterial strains (Table 3). MIC values of leaves were not significantly (*p* > 0.01) different from pods and bark extracts but MBC values of leaves were significantly (*p* < 0.01) different compared to pods and bark. The MIC values of acacia leaves and bark extracts against multidrug-resistant *E. coli* were lower in the current study than previously reported for *E. coli*, causing otitis infection [22]. The leaves and bark extracts in the current study also showed lower MIC value against *Salmonella typhimurium* than previously reported for *Salmonella typhi* [21]. The reported differences might be due to different strains of *E. coli* and *Salmonella*, and/or different experimental protocols.

The antibacterial activity of ethanol extracts of leaves, pods and bark of *A. nilotica* against drug sensitive and multidrug-resistant *E. coli* and *Salmonella* spp. obtained from clinical and food sources were examined. All parts of plant were found to be effective even against the clinical isolates of *E. coli* and *Salmonella typhimurium* that were resistant to various commercially available antibiotics. Therefore, acacia can be an alternate approach to treat resistant pathogens either in the form of its purified extract or in combination with commercially available antibiotics. Antibacterial activity determined by the disc diffusion method has certain limitations since this assay indicates only growth inhibition of bacteria without any evidence that the tested extracts are either bacteriostatic or bactericidal. Therefore, MIC and MBC values of extracts were determined to specify the dose and nature of the activity. However, more research is needed to validate these findings in animal models and eventually in human clinical trials.

### 2.5. Kill-Time Analysis

**Figure 2:**
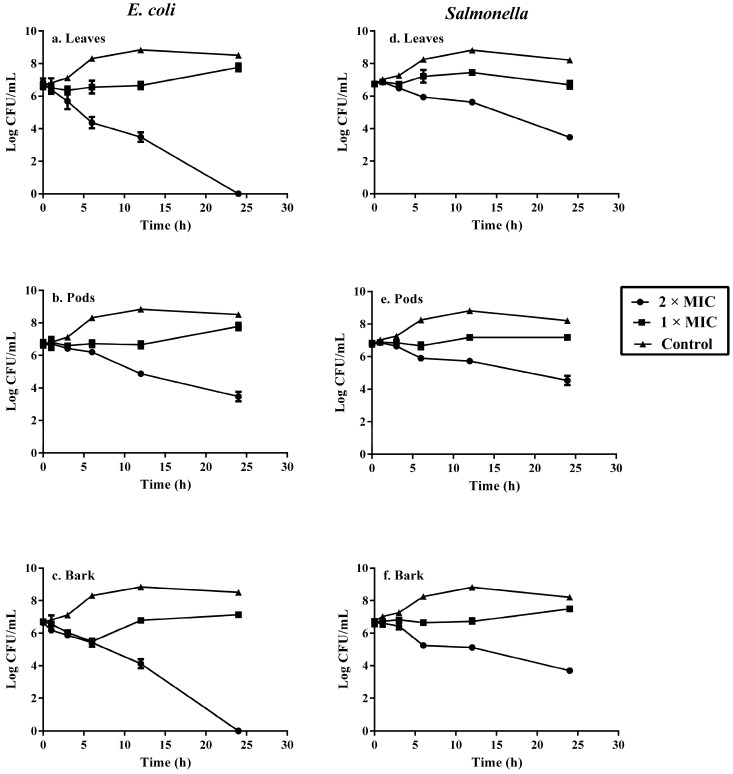
Effects of acacia leaves, pods and bark extracts on the viability of tested *E. coli* (**a**–**c**) and *Salmonella typhimurium* (**d**–**f**).

The kill-time analysis of clinical strains of *E. coli* and *salmonella* was performed by evaluating the decrease in CFU/mL with time at the following concentrations of acacia extracts, control, 1 × MIC and 2 × MIC, respectively. The tested pathogens showed decreased viability when exposed to acacia extracts. The effects of leaves, pods and bark extracts on viability of *E. coli* are shown in Figure 2. During the first five hours, there was a decreased number of *E. coli* when treated with MIC and later there was a slower growth as compared to the control that clearly indicated that MIC of leaves, pods and bark inhibit the growth of bacteria. The leaves and bark extracts at a concentration of 2 × MIC killed all the bacterial cells by the end of 24 h.

The effects of leaves, pods and bark extracts on the viability of *Salmonella typhimurium* are shown in Figure 2. The MIC of all extracts inhibited the growth of bacteria over a period of 24 h as compared to control, whereas the 2 × MIC showed a declined viability of test bacteria from 6.75 to 3.47 Log CFU/mL for leaves, 6.75 to 4.54 Log CFU/mL for pods and 6.65 to 3.70 Log CFU/mL for acacia bark.

The extracts of acacia showed variable kinetics against tested pathogens. The concentration-dependent killing showed bacteriostatic and bactericidal effects of extracts. Test compounds were considered bacteriostatic at the lowest concentration that reduced the original inoculum size by 0–3 Log CFU/mL and bactericidal if inoculum size was reduced by >3 Log CFU/mL [23]. Complete elimination of *E. coli* was observed after 24 h of treatment with leaves and bark extracts at concentration of 2 × MIC. This supported previous research that reported 99.9% reduction in the growth of *Salmonella typhi* in an *A. nilotica* time-kill assay [24]. The pods were able to decrease the inoculum size from 6.62 to 3.47 Log CFU/mL after 24 h at a concentration of 2 × MIC.

### 2.6. Bacterial Cell Membrane Permeability after Treatment with Acacia Extracts

**Figure 3:**
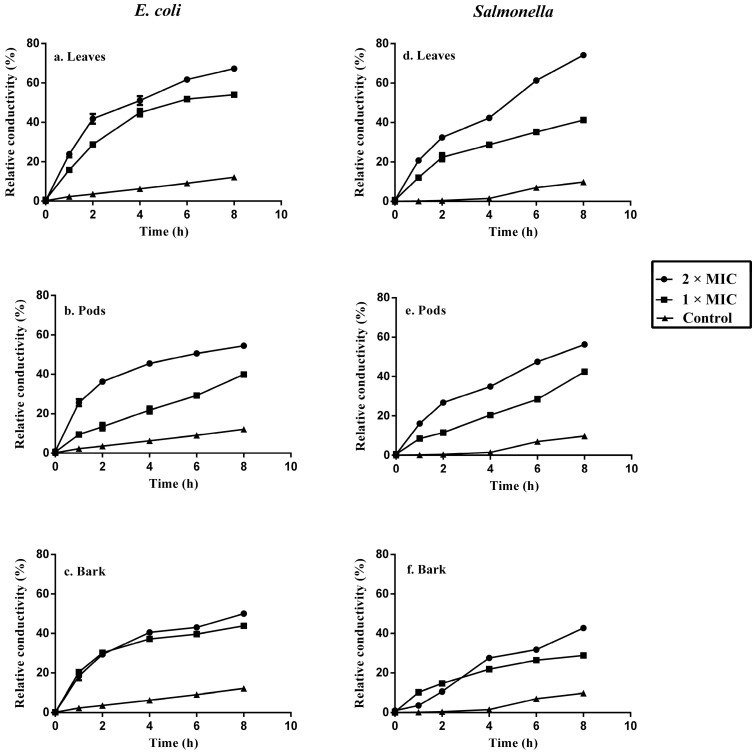
Effects of acacia leaves, pods and bark extracts on the impermeability of cell membrane of tested *E. coli* (**a**–**c**) and *Salmonella typhimurium* (**d**–**f**).

Bacterial cell membrane permeability was determined in terms of relative electric conductivity. Evaluation of the relative electric conductivity of bacterial cells treated with plant extracts demonstrated that leaves, pods and bark extracts have an effect on the membrane permeability of *E. coli* and *Salmonella typhimurium*. All plant extracts resulted in increased relative electrical conductivity of bacterial cells, which indicated a leakage of intracellular ingredients especially electrolytes from the cells. The leaves of acacia induced maximum relative permeability of 67.25% ± 0.82% and 74.19% ± 0.72% at concentration of 2 × MIC for *E. coli* and *Salmonella typhimurium*, respectively, that was more than pods and bark extracts as shown in Figure 3. Maintaining ion homeostasis is integral to the maintenance of energy status, solute transport, metabolic regulation, control of turgor pressure and motility of cell, therefore a slight change of the structural integrity of the cell membrane can affect metabolism and lead to cell death [25].

The results in this study showed that bacterial cell membrane permeability changed with increasing concentrations of extracts and incubation time period, which caused leakage of intracellular electrolytes. Similar results were reported by Zhao et al. [26], showing an increase in electric conductivity of bacterial cells with increasing concentrations of sugarcane bagasse extracts. There was a modest change in relative electrical conductivity of the control incubation during the first four hours. A rise in relative electrical conductivity after four hours was found due to normal lysis and bacterial death resulting in increased relative electrical conductivity.

### 2.7. Integrity of Bacterial Cell Membrane

The integrity of the cell membrane was determined by treating the bacterial cells with different concentrations of plant extracts (control, 1 × MIC and 2 × MIC) and measuring the released cell constituents, including proteins and absorbance at 260 nm of the supernatant of tested bacteria. The effects of different concentrations of each extract on tested bacteria are shown in Table 4. The results indicated that there was significant increase (*p* < 0.01) in the release of cellular constituents and protein concentration with increasing concentrations of acacia extracts. These results corroborate previous reports, indicating that irreversible damage might occur to bacterial membranes after treatment with plants, which could lead to loss of essential cellular components such as proteins and nucleic acids [27]. The leaves showed higher protein and nucleic acid released contents as compared to pods and bark at concentrations of 1× MIC and 2 × MIC. The results indicated that tested extracts cause irreversible damage to the cell membrane, which led to loss of cellular constituents and finally to cell death.

The macromolecules of microbial cells, including nucleic acids and proteins, which constitute key structural components, were released from the tested bacteria after treatment with leaves, pods and bark extracts of acacia. The measurements of specific leakage markers after treatment with extracts, including nucleic acids and protein, is an indicator of bacterial cell membrane integrity in comparison to unexposed cells [28]. The results from this study indicated rapid loss of proteins and nucleic acids from treated pathogens, due to irreversible damage to the cytoplasmic membranes. *A. nilotica* is rich in phenolic compounds [29] and phenolic acids can cause irreversible changes to cell membrane [30]. Thus integrity of cell membrane was concluded as an important factor to inhibit the pathogenic bacterial growth. Further research is needed to find the target damage sites on bacteria cells to make sure that either antimicrobial effect was from damage to lipopolysaccharide or membrane proteins in cell wall.

### 2.8. Scanning Electron Microscope Observations

**Figure 4:**
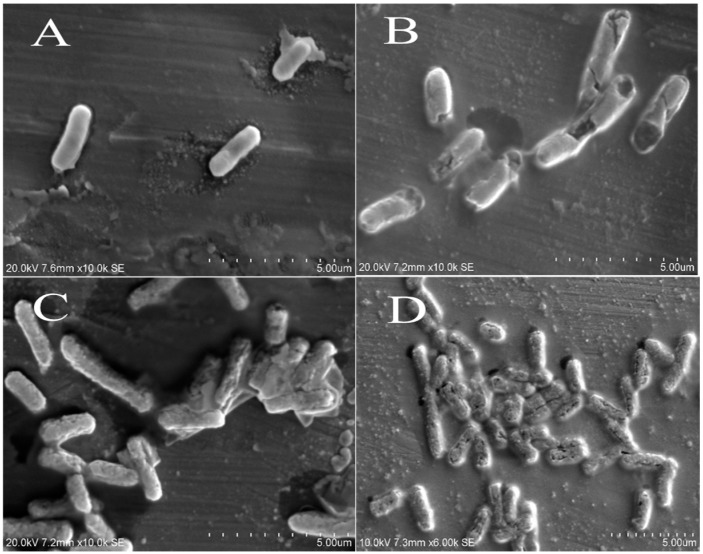
Scanning electron microscope imaging of untreated bacterial cells of (**A**) *E. coli* (clinical isolates); treated bacterial cells of *E. coli* (clinical isolate) with (**B**) leaves; (**C**) pods; (**D**) bark extracts of acacia.

**Figure 5:**
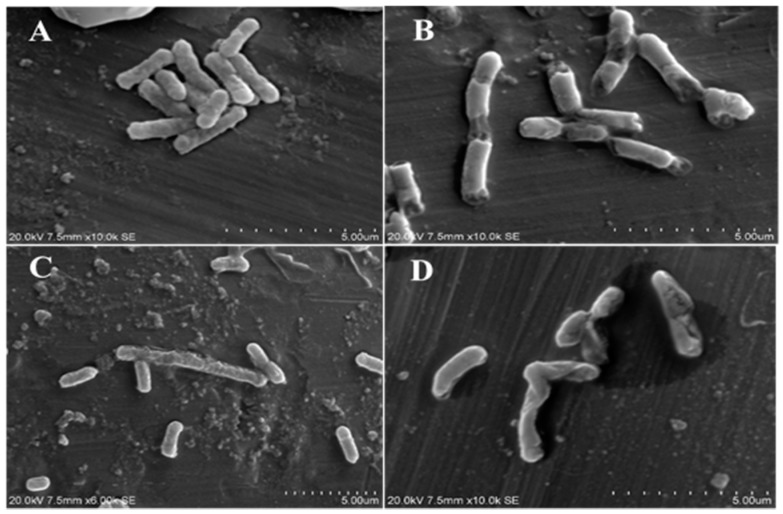
Scanning electron microscope imaging of untreated bacterial cells of (**A**) *Salmonella typhimurium* (clinical isolates); treated bacterial cells of *Salmonella typhimurium* (clinical isolate) with (**B**) leaves; (**C**) pods; (**D**) bark extracts of acacia.

To investigate the antibacterial mode of action, it is essential to evaluate changes in bacterial cell membrane permeability, integrity, morphology and surface characteristics [27]. The physiological and morphological changes in *E. coli* (Figure 4) and *Salmonella typhimurium* (Figure 5) were observed by scanning electron microscope (SEM) after treatment with acacia extracts. Results showed a directly destructive effect of acacia extracts on tested pathogens. The treated bacterial cells showed obvious morphological changes as compared to untreated cells. Most of treated bacterial cells became pitted, deformed and broken. These observations supported the results of cell permeability and integrity assay, and indicated that extracts of *A. nilotica* had major effects on the cell wall and cytoplasmic membrane of bacteria. 

## 3. Material and Methods

### 3.1. Preparation of Plant Extracts and Microbial Sample Collection and Identification

Leaves, pods and bark of the *A. nilotica* plant (wild) were collected from Lahore, Pakistan. The plant was authenticated and voucher specimen (Voucher number S6 HbGCS with Reference number 7998) was deposited to Botany Society Government College of Science, Lahore, Pakistan. The selected plant parts were washed thoroughly under running tap water to remove the surface dirt, followed by rinsing with sterilized distilled water. The plant samples were dried under shade in open air for 48 h. The dried samples were grounded by means of a mechanical grinder (Philips Co. Ltd., Shanghai, China) and finally into finely divided powder by pestle and mortar.

The extraction from these dried parts of *A. nilotica* was conducted following the method as described by Adwan et al. [31], with slight modifications. Powdered plant samples (30 g) were placed in 250 mL of ethanol (80%, *v/v*) in conical flasks and placed on shaking incubator (Gallenkamp, UK) at 200 rpm for 48 h at 25 °C. The extracts were filtered and concentrated by means of a rotary evaporator (Büchi rotavapor R-144, Flawil, Switzerland) followed by lyophilization for 24 h in a freeze dryer (Scanvac Cool Safe 55-4, Scanvac, Denmark). The freeze dried extracts were stored at 4 °C until further use.

*E. coli*, *Salmonella enterica* subsp. *enterica, Salmonella enteritidis* and *Salmonella typhimurium* were isolated from beef and poultry meat samples and identified by biochemical and immunological testing in the Bioprocess Technology laboratory at the Asian Institute of Technology, Bangkok, Thailand. Other human clinical isolates, namely *E. coli* and *Salmonella typhimurium* were acquired from the clinical laboratory of Thammasat Hospital, Pathumthani, Thailand.

### 3.2. Antibiogram of Salmonella spp. and E. coli

Antibacterial susceptibility patterns of *Salmonella* spp. and *E. coli* isolates were determined by using the disc diffusion assay, following the guidelines of CLSI, M100-S23 [32]. The following commonly used antibiotics were tested: ampicillin (10 μg), amoxicillin (10 μg), chloramphenicol (30 μg), gentamicin (10 μg), amikacin (30 μg), streptomycin (10 μg), tetracycline (30 μg), trimethoprim–sulfamethoxazole (25 μg), ciprofloxacin (5 μg), and ceftriaxone (30 μg) (Oxoid, UK). The pre-incubated 24 h cultures of *E. coli* and *Salmonella* were adjusted to 0.5 McFarland standard to get 10^8^ CFU/mL. The bacterial suspensions were spread by using the sterilized cotton swab on the surface of Mueller-Hinton Agar (MHA) (Himedia, India) plates. Antibiotic discs were dispensed by using sterilized forceps on the surface of agar medium and gently pressed. The plates were then incubated for 24 h at 37 °C and results were measured as diameter of inhibition zone in triplicates.

### 3.3. Detection of Beta-Lactam- and Tetracycline-Resistant Genes

*E. coli* and *Salmonella* isolates were sub-cultured overnight in nutrient broth and genomic DNA was extracted by using genomic DNA purification kit (Insta-max gene matrix Bio-Rad, Hercules, CA, USA) according to manufacturer’s instructions. The presence of genes associated with resistance to tetracycline, *tet (A)*, *tet(B*) and beta lactams (*bla _SHV_*, *bla _CMY_*) were determined by PCR and set of primers used for each gene were acquired from Sigma Aldrich, Singapore (Table 5). PCR reactions were performed in a total volume of 25 µL including, 14 µL PCR master mix (Tag PCR master mix kit, Bio-Rad, Hercules, CA, USA), 9 µL of DNA (50–200 ng/µL) and 1 µL of each reverse and forward primer (10 μM). PCR reactions were carried out by using DNA thermos-cycler (Bio-Rad) as follows: initial denaturation for 30 s at 95 °C followed by 30 cycles, for 30 s at 95 °C, annealing for 30 s (65.2, 57.2, 61.1 and 60.3 °C for *bla _CMY_*, *bla _SHV_*, *tet (A)* and *tet (B)*, respectively) and 1 min at 68 °C, followed by a final extension step of 5 min at 68 °C. Amplified samples were subjected to gel electrophoresis by using QIAxcel advance system (QIAGEN Inc., Valencia, CA, USA) and results were interpreted by QIAxcel screenGel 1.4.0.

### 3.4. Antimicrobial Activity of Acacia Extracts

The antibacterial effects of acacia extracts were determined by a method as described by Duraipandiyan, Ayyanar and Ignacimuthu [35] with slight modifications. Each inoculum was adjusted to 0.5 McFarland standard and spread with the help of a sterilized cotton swab on the surface of Muller Hilton agar plates. The different concentrations of extracts (5 and 10 mg/disc) were loaded on 6 mm sterile discs and discs were placed on the surface of agar medium. Amikacin (30 µg/mL) was used as a positive control, whereas dimethyl sulfoxide (DMSO) was used as negative control. The plates were incubated for 24 h at 37 °C. The results were read by measuring the diameter of clear zone around the discs. All experiments were conducted in triplicates.

### 3.5. Determination of Minimum Inhibitory Concentration and Minimum Bactericide Concentration

MIC and MBC were determined according to a method described by Kubo et al. [36], with some modifications. Stock solution of each extract was prepared in DMSO. Two-fold serial dilutions of extracts were filtered through 0.45 µm millipore filters and prepared in sterile nutrient broth (Himedia, India) to obtain the concentrations of 25, 12.5, 6.25, 3.12, 1.56, 0.78 and 0.39 mg/mL. Each inoculum was adjusted to 0.5 McFarland standard and then diluted to 1:100 for broth dilution method. The standardized inoculum was introduced in each concentration of extract. The test tubes containing solvent blank in nutrient broth along with standardize inoculum were used as growth control. The test tubes containing broth without inoculum and extract were used as negative control. The test tubes were further incubated for 24 h at 37 °C in the incubator and the lowest concentration that had no visible growth after 24 h incubation was considered as MIC. MBC was determined by sub-culturing all concentrations that had no detectable growth. 100 µL from each dilution was inoculated on the surface of freshly prepared nutrient agar (Himedia, India) plates and incubated for 24 h at 37 °C. The minimum concentration that had no visible growth on agar plates after 24 h incubation was considered as MBC. Each experiment was conducted in triplicates.

### 3.6. Kill-Time Analysis

Kill-time curve analysis was used to estimate the bactericidal effects of leaves, pods and bark extracts of acacia plant, according to a method as described by Joray et al. [37], with slight modifications. The stock solutions of plant extracts were prepared in DMSO and different concentrations (MIC and 2 × MIC) of extracts were prepared in nutrient broth. The hospital acquired multidrug-resistant strains of *E. coli* and *Salmonella typhimurium* were selected for kill-time curve analysis. The test bacteria (1 × 10^8^ CFU/mL) were added to each concentration of plant extract. The cultivation of bacteria with MIC, 2 × MIC and control (DMSO) were done simultaneously. At selected time intervals (0, 1, 3, 6, 12 and 24 h), samples were taken from tested bacterial culture, serially diluted in sterile water and incubated in Plate Count Agar. The CFU/mL were counted after incubation at 37 °C for 24 h.

### 3.7. Bacterial Cell Membrane Permeability

The permeability of bacterial cell membrane was determined by a method described by Kong et al. [38], with slight modifications. The pathogenic strains of *E. coli* and *Salmonella typhimurium* (human clinical isolates) were incubated at 37 °C for 10 h in nutrient broth. After incubation, bacterial cells were separated by centrifugation at 1500× *g* for 10 min. The glucose solution (5 %, *w/v*) was used to wash the bacteria repeatedly, until electrical conductivities of bacterial cells were near to 5 % glucose, as in the case of isotonic bacterial cells. The extracts of acacia at two different concentrations (MIC and 2 × MIC) were added to glucose (5 %, *w/v*) separately and after mixing properly, electrical conductivities were determined and marked as L_1_, followed by addition of different concentrations of extracts to isotonic bacterial glucose solution. After mixing, the samples were incubated at 37 °C for 8 h and conductivities were measured at various time intervals (0, 1, 2, 4, 6 and 8 h) and marked as L_2_.

The conductivity of glucose (5 %, *w/v*) treated bacterial cells in boiling water for 5 min was taken as control and marked as L_o_. The change in bacterial cell membrane permeability was calculated in terms of relative electric conductivity (%) by using the following Equation:

Relative electric conductivity (%) = 100 × (L_2_ − L_1_)/L_o_

### 3.8. Integrity of Bacterial Cell Membrane

The integrity of the bacterial cell membrane was determined by the method described by Du et al. [39], with slight modifications. The working culture (100 mL) of tested bacteria was centrifuged at 3000× *g* for 15 min. The cells were collected, washed three times and re-suspended in 0.1 M phosphate buffer solution (PBS, pH 7.4). The PBS buffer (100 mL) treated bacterial culture was incubated with different concentration of plant extracts (control, MIC and 2 × MIC) at 37 °C for 6 h under agitation. Then 25 mL of samples were collected and centrifuged at 11,000× *g* for 5 min. The protein concentrations in the supernatants were determined by the Bradford assay [40]. The concentrations of released cellular constituents mainly comprised of nucleic acids were determined by using 3 mL of supernatant and measuring absorption at 260 nm by UV-visible spectrophotometer (UNICAM UV/Vis Spectrophotometer, Cambridge, UK). The absorption of PBS containing same concentrations of extract after 2 min contact with tested bacteria was used for correction. The untreated cells were corrected with PBS.

### 3.9. Scanning Electron Microscope Observations

The efficacy of acacia extracts and morphological changes of *E. coli* and *Salmonella* strains were observed by SEM microscopy. The tested bacterial cells were incubated in nutrient broth at 37 °C for 10 h. The bacterial cells were then treated with determined MIC values of acacia extracts, (control culture was left untreated). After incubation period of 6 h at 37 °C, bacterial cells were harvested by centrifugation at 1500× *g* for 10 min. The precipitated cells were washed 3 times and re-suspended in 0.1 M PBS (pH 7.4). The bacterial suspensions (20 µL) were spread onto a microscopic slide and air dried. The samples were coated with gold particles under vacuum followed by microscopic examination by using SEM (Hitachi S-3400N, Tokyo, Japan).

### 3.10. Statistical Analysis

All experiments were carried out in triplicates and results are expressed as mean values with standard deviation (±SD). One-way analysis of variance (ANOVA) was carried out to determine significant differences (*p* < 0.01 were considered statistically significant) between means by using SPSS statistical software package (SPSS, version 16.0, SPSS Inc., Chicago, IL, USA).

## 4. Conclusions

Consumers are increasingly demanding safe, natural and high quality food products. The trend of using natural antimicrobials is becoming an attractive approach in the field of food preservation and safety because synthetic antimicrobials might be associated with various health hazards. Medicinal plants, such as *A. nilotica*, could be an alternative approach because of their safety, relatively low cost and effectiveness against multidrug-resistant pathogens. The current study investigated *A. nilotica* extracts as a natural antimicrobial agent and elucidated the mode of action on food source (food spoilage) and clinical (pathogenic) isolates of *E. coli* and *Salmonella*. Acacia extracts showed substantial antimicrobial effects against antibiotic-resistant bacterial strains, by observing changes in bacterial cell morphology and cell membrane integrity and permeability. Further purification, isolation and identification are needed to develop novel antimicrobial compounds from *A. nilotica* for food and pharmaceutical applications.

## Figures and Tables

**Table 1:** Antibiogram of *E. coli* and *Salmonella* isolates.

Bacteria	Resistance	Susceptible
E1	Amp, Aml, Chl, Gen, Tet, Cip, Ctx	Amk, Str, Sxt
E2		Amp, Aml, Chl, Gen, Amk, Str, Tet, Sxt, Cip, Ctx
E3	Amp, Aml, Chl, Tet	Gen, Amk, Str, Sxt, Cip, Ctx
S1	Amp, Aml, Str, Tet	Chl, Gen, Amk, Sxt, Cip, Ctx
S2		Amp, Aml, Chl, Gen, Amk, Str, Tet, Sxt, Cip, Ctx
S3	Tet, Chl	Amp, Aml, Gen, Amk, Str, Sxt, Cip, Ctx
S4	Amp, Aml, Tet	Gen, Amk, Str, Cip, Ctx, Chl, Sxt

Amp = Ampicillin, Aml = Amoxicillin, Chl = Chloramphenicol, Gen = Gentamycin, Tet = Tetracycline, Cip = Ciprofloxacin, Amk = Amikacin, Str = Streptomycin, Sxt = Trimethoprin/Sulfamethoxazole. E1 = *E. coli* and S1 = *Salmonella typhimurium* were isolated from clinical samples, E2 = *E. coli*, S2 = *Salmonella enterica* and S3 = *Salmonella typhimurium* were isolated from poultry meat, and E3 = *E. coli* and S4 = *Salmonella enteritidis* were isolated from beef meat samples.

**Table 2:** Antibacterial effects of *Acacia nilotica* extracts against clinical and food isolates of *E. coli* and *Salmonella*.

Sample	Conc. (mg/disc)	Zone of Inhibition (mm)								
		E1	E2	E3	Mean of *E. coli* Strains	S1	S2	S3	S4	Mean of *Salmonella* Strains
Leaves	5	17.0 ± 1.0	16.7 ± 1.15	16.7 ± 0.58	16.78 ± 0.83 ^a^	12.3 ± 0.58	11.3 ± 1.53	11.3 ± 2.08	11.7 ± 1.15	11.67 ± 1.30 ^a^
	10	21.7 ± 0.6	21.0 ± 1.0	20.7 ± 1.53	21.11 ± 1.05 ^b^	17.7 ± 0.58	16.7 ± 0.58	16.0 ± 1.0	17.0 ± 1.0	16.83 ± 0.94 ^b^
Pods	5	9.3 ± 0.58	8.3 ± 0.58	7.7 ± 0.58	8.44 ± 0.88	8.3 ± 0.58	8.7 ± 1.15	7.7 ± 1.15	8.3 ± 0.58	8.25 ± 0.86
	10	19.0 ± 1.0	18.3 ± 0.58	15.3 ± 0.58	17.56 ± 1.81 ^b^	15.7 ± 1.53	13.7 ± 0.58	14.7 ± 1.15	15.0 ± 1.0	14.75 ± 1.21 ^b^
Bark	5	8.7 ± 0.58	9.0 ± 1.0	8.3 ± 0.58	8.67 ± 0.71	5.3 ± 4.61	8.7 ± 0.58	8.7 ± 1.15	8.0 ± 1.0	7.67 ± 2.53
	10	13.0 ± 1.0	12.3 ± 0.58	11.3 ± 1.53	12.22 ± 1.20 ^b^	10.3 ± 1.15	11.3 ± 1.15	11.7 ± 0.58	11.0 ± 2.0	11.08 ± 1.24 ^b^
Amikacin (Control)	30 µg	22.4 ± 1.51	23.8 ± 1.73	22.5 ± 1.1	22.8 ± 1.43	23.4 ± 2.14	23.5 ± 1.53	22.0 ± 1.0	23.0 ± 1.0	22.97 ± 1.24

The results were expressed as mean ± S.D of triplicates. Superscript “a” represents means that are statistically different (*p* < 0.01) compared to all other extracts at concentration of 5 mg/disc against *E. coli* and *Salmonella* strains. Superscript “b” represents means that are statistically different (*p* < 0.01) between all extracts at concentration of 10 mg/disc against *E. coli and Salmonella* strains. E1 = *E. coli* and S1 = *Salmonella typhimurium* were isolated from clinical samples, E2 = *E. coli*, S2 = *Salmonella enterica* and S3 = *Salmonella typhimurium* were isolated from poultry meat, and E3 = *E. coli* and S4 = *Salmonella enteritidis* were isolated from beef meat samples.

**Table 3:** Minimum inhibitory concentration (MIC) and minimum bactericidal concentration (MBC) of *A. nilotica* extracts.

Microbial Strains	Minimum Inhibitory Concentration			Minimum Bactericide Concentration		
	Leaves	Pods	Bark	Leaves	Pods	Bark
	(mg/mL)	(mg/mL)	(mg/mL)	(mg/mL)	(mg/mL)	(mg/mL)
E1	3.12	3.12	6.25	6.25	12.5	12.5
E2	3.12	6.25	6.25	3.12	12.5	12.5
E3	3.12	3.12	6.25	6.25	12.5	12.5
S1	1.56	3.12	3.12	3.12	6.25	6.25
S2	1.56	3.12	3.12	3.12	6.25	6.25
S3	1.56	3.12	3.12	3.12	6.25	12.5
S4	1.56	3.12	3.12	3.12	6.25	6.25

The results were expressed as mean of triplicates. E1 = *E. coli* and S1 = *Salmonella typhimurium* were isolated from clinical samples, E2 = *E. coli*, S2 = *Salmonella enterica* and S3 = *Salmonella typhimurium* were isolated from poultry meat, and E3 = *E. coli* and S4 = *Salmonella enteritidis* were isolated from beef meat samples.

**Table 4:** Effects of acacia leaves, pods and bark extracts on cell constituents’ release of tested *E. coli* and *Salmonella typhimurium*.

Sample	Conc.	Cell Constituents Release			
		*E. coli*		*Salmonella typhimurium*	
		Protein (µg/mL)	Cell Constituents (OD_260nm_)	Protein (µg/mL)	Cell Constituents (OD_260nm_)
Acacia leaves	2 × MIC	62.70 ± 4.20	0.42 ± 0.007	48.26 ± 5.25	0.4 ± 0.006
	1 × MIC	32.33 ± 4.00	0.34 ± 0.007	25.67 ± 4.84	0.31 ± 0.001
	Control	8.63 ± 2.31	0.086 ± 0.013	10.11 ± 2.22	0.09 ± 0.004
Acacia pods	2 × MIC	30.85 ± 2.80	0.31 ± 0.029	18.63 ± 2.31	0.27 ± 0.01
	1 × MIC	17.52 ± 2.31	0.23 ± 0.036	12.33 ± 2.22	0.164 ± 0.025
	Control	6.78 ± 2.94	0.102 ± 0.006	5.67 ± 2.94	0.1 ± 0.017
Acacia bark	2 × MIC	39.74 ± 4.62	0.30 ± 0.013	31.96 ± 3.90	0.29 ± 0.006
	1 × MIC	22.33 ± 2.94	0.18 ± 0.016	18.67 ± 1.64	0.14 ± 0.009
	Control	5.67 ± 2.22	0.088 ± 0.011	7.18 ± 1.65	0.1 ± 0.01

The results were expressed as mean ± S.D. of triplicates. MIC = minimum inhibitory concentration.

**Table 5:** Beta-lactams- and tetracycline-resistant genes and primer sequences used for polymerase chain reaction.

Antimicrobial Agent	Resistant Gene	Sequence	Size (bp)	Annealing Temp (°C)	Reference
Beta-Lactams	*bla _CMY_* F	TGGCCAGAACTGACAGGCAAA	462	65.2	[33]
	*bla _CMY_* R	TTTCTCCTGAACGTGGCTGGC			
	*bla _SHV_* F	TCGCCTGTGTATTATCTCCC	768	57.2	[33]
	*bla _SHV_* R	CGCAGATAAATCACCACAATG			
Tetracycline	*tet (A)* F	GGTTCACTCGAACGACGTCA	577	61.1	[34]
	*tet (A)* R	CTGTCCGACAAGTTGCATGA			
	*tet (B)* F	CCTCAGCTTCTCAACGCGTG	634	60.3	[34]
	*tet (B)* R	GCACCTTGCTGATGACTCTT			

*bla _CMY_* and *bla _SHV_* present beta-lactams-resistant genes and *tet (A)* and *tet (B)* present tetracycline-resistant gene sequences, F = forward primer and R = reverse primer and bp = base pairs.
